# Impact of bile salts, biopolymer coatings, and food matrix on bilosome-mediated delivery of trans-resveratrol^☆^

**DOI:** 10.1016/j.fochx.2026.103547

**Published:** 2026-01-16

**Authors:** Aygul Can, Taskeen Niaz, Arwen I.I. Tyler, Alan R. Mackie

**Affiliations:** a School of Food Science and Nutrition, University of Leeds, Leeds, LS2 9JT, UK; b The Department of Gastronomy and Culinary Arts, Faculty of Architecture, Design and Fine Arts, Islam Science and Technology University, Gaziantep, 27010, Türkiye

**Keywords:** Bilosome, Trans-resveratrol, Chitosan, Polygalacturonic acid, Oat milk, Bioaccessibility, Absorption

## Abstract

This study compared liposomes, bilosomes, and biopolymer-coated bilosomes for trans-resveratrol (*t*-res) delivery, evaluating the effects of bile salts, biopolymer coatings, and oat milk (OM) on digestion behaviour, bioaccessibility, and intestinal absorption. The bioaccessibility of *t*-res increased by 1.82-fold with liposomes and by 2.14–2.32-fold with bilosomes. *Ex vivo* absorption studies using murine intestinal tissue confirmed enhanced uptake and tissue accumulation with bilosomes, reaching 16.801 μM compared to 0.400 μM for liposomes. Coating bilosomes with chitosan (CH) and polygalacturonic acid (PGA) improved stability and surface properties but reduced bioaccessibility to 8–40%, depending on biopolymer concentration. The OM influenced both initial and digestion-related properties, enhancing bioaccessibility in uncoated systems while reducing intestinal tissue accumulation of *t*-res. Overall, these findings indicate that bilosomes outperform liposomes for hydrophobic compound delivery; however, their performance is highly formulation-dependent and strongly influenced by food matrix interactions, underscoring the importance of consumption context in functional food design.

## Highlights

• Bilosome behaviour was evaluated for the first time in a real food matrix.
• *t*-res bioaccessibility reached 90% with bilosomes *vs* 70% with liposomes.
• Biopolymer-coated bilosomes showed 8–40% *t*-res bioaccessibility.
• Oat milk increased *t*-res bioaccessibility ∼1.2-fold in liposomes and bilosomes.
• Bilosomes enabled intestine-targeted oral delivery and improved *t*-res absorption.

## Graphical abstract

**Figure:**
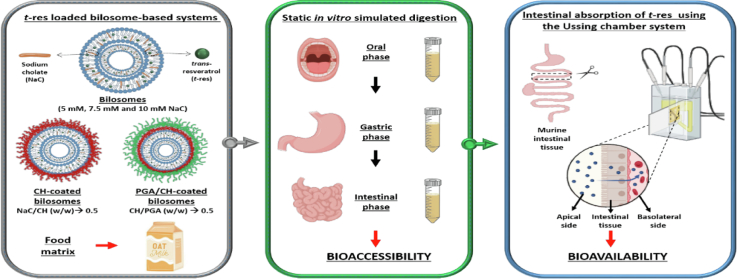

## 1 Introduction

Phenolic compound properties are influenced by their chemical structure, molecular size, solubility, and permeability (Chimento et al., 2019). Among such compounds, tran*s*-resveratrol (*t*-res) is widely studied for its antioxidant, antimicrobial, anti-inflammatory, and anti-carcinogenic properties (Can et al., 2021). However, its very low aqueous solubility (<0.001 mol/L) and rapid metabolism in the small intestine to glucuronide and sulphate conjugates severely limit its oral bioavailability, which remains below 1% (Brown et al., 2024; Drago et al., 2024). Both unmetabolised resveratrol and its metabolites are quickly cleared from circulation, preventing effective concentrations being achieved at target sites. While some reabsorption occurs *via* enterohepatic recirculation, overall systemic exposure remains minimal. Therefore, encapsulation in lipid-based delivery systems has been proposed to enhance solubility, protect against enzymatic and chemical degradation, and control release.

Bile salt-containing liposomes have been referred to as bilosomes (Can et al., 2021; Mitrović et al., 2025; Obeid et al., 2025), ultra-deformable liposomes (Arora et al., 2020; Barone et al., 2020), or elastic liposomes (Wu et al., 2019). Bilosomes are based on liposomal structures and can encapsulate both hydrophobic and hydrophilic bioactive compounds (Van Tran et al., 2019), offering advantages due to the physiological role of bile salts in digestion and nutrient absorption. During digestion, ∼95% of bile salts are reabsorbed and recycled, with the rest excreted (Bauer et al., 2005). Beyond digestion, bile salts have been used in pharmaceutical, cosmetic, and chemical applications (Abdel-moneum & Abdel-Rashid, 2023; Donkers et al., 2019; Elnaggar, 2015) and supplementation studies in animals suggest they can modulate lipid metabolism depending on dose, bile salt type, dietary lipid content, and gut microbiota composition (Ding et al., 2020; Jiang et al., 2018; Liao et al., 2020; Liu, Azad et al., 2022; Wang et al., 2024).

Bile salts, as biological surfactants, can induce curvature in phospholipid bilayers in a concentration-dependent manner, promoting structural transitions (Garidel et al., 2007) that alter loading capacity (LC%) and result in different bioaccessible concentrations (Lichtenberg et al., 2013; McClements, 2013) compared to other lipid-based delivery systems, including those based on liposomes. Incorporating bile salts into phospholipid bilayers can enhance encapsulation efficiency (EE%) and LC%, increase bioaccessibility, improve gastrointestinal stability, boost therapeutic efficacy, and promote greater biocompatibility (Elebyary et al., 2025; Jeong et al., 2025; Liu, Guo et al., 2022; Milia et al., 2025; Wang et al., 2025). The amphiphilic ionic nature of bile salts allows them to buffer acidic conditions through the protonation of carboxyl groups and increase bilayer elasticity and permeability, thereby improving bilosome-mediated absorption (Abdel-moneum & Abdel-Rashid, 2023; Jeong et al., 2025; Obeid et al., 2025; Tang et al., 2021). While these properties make bilosomes promising carriers for hydrophobic bioactives, their application in food fortification has been limited. This is largely due to the distinctly bitter taste attributed to bile salts (Heaton & Morris, 1971; Ziegler et al., 2023), which can impair consumer acceptance.

Taste masking is therefore essential for practical use in foods or oral supplements. One strategy involves coating bilosomes with biopolymers such as chitosan (CH), polygalacturonic acid (PGA), or pectin. These coatings can shield unpleasant flavours, improve stability, and modulate gastrointestinal behaviour. CH has been used to encapsulate bitter compounds and reduce perceived bitterness (Liu et al., 2017; Rajesh et al., 2015). PGA and pectin have also demonstrated taste-masking capabilities for various bioactives (Ahmed et al., 2024; Huang et al., 2024; Müller et al., 2018). Beyond sensory benefits, these coatings can influence digestion, release, and absorption kinetics (Alhakamy et al., 2021; Hegazy et al., 2022; Kengkittipat et al., 2021).

Plant-based milk alternatives have gained increasing attention due to rising health and sustainability concerns, including lactose intolerance, food allergies, and the environmental impacts associated with dairy production (Cui et al., 2023). Among these alternatives, oat milk (OM) has rapidly achieved global popularity owing to its smooth texture, mild flavour, and high consumer acceptance (Yu et al., 2023). Oats represent a sustainable dietary source rich in nutrients and phytochemicals, providing functional proteins, lipids, dietary fibre, vitamins, minerals, and various bioactive compounds (Li et al., 2022). Oat milk, produced from whole-grain oats, constitutes a complex colloidal food system containing oil droplets with a triglyceride core surrounded by a phospholipid–protein shell (Nikiforidis, 2019). Due to its widespread consumption, nutritional value, and representative structural complexity, oat milk was selected as an appropriate model food matrix for evaluating the behaviour and bioaccessibility of functional ingredient carriers.

This study investigates bilosomes as carriers for *t*-res in food fortification, focusing on how the bile salt sodium cholate (NaC, biopolymer coatings (CH, PGA), and the presence of oat milk (OM) influence gastrointestinal stability, bioaccessibility, and absorption. Despite the growing interest in bilosome-based delivery systems for enhancing the oral bioavailability of hydrophobic phytochemicals, existing research has largely focused on pharmaceutical formulations employing uncoated (Abbas et al., 2021; Said et al., 2024) or biopolymer-coated bilosomes (Alhakamy et al., 2021; Hegazy et al., 2022; Kengkittipat et al., 2021), whereas their performance under food-relevant conditions remains largely unexplored (Jeong et al., 2025; Liu, Guo et al., 2022; Wang et al., 2021). In particular, the combined and comparative effects of bile salts and biopolymer coatings on behaviour in the gastrointestinal environment, bioaccessibility, and site-specific delivery during digestion are still poorly understood. To address this gap, the present study systematically compared liposomes, bilosomes, and biopolymer-coated bilosomes for t-res delivery using a standardised *in vitro* digestion model in the presence of OM as a real food matrix. By simultaneously evaluating digestion behaviour, bioaccessibility, and absorption, this work provides new insights into the rational design of bilosome-based lipid delivery systems tailored for small-intestinal targeting in functional food and nutraceutical applications. Notably, these findings reveal how delivery system composition and matrix interactions jointly determine *t*-res performance and provide the first in-depth assessment of bilosome behaviour within a food matrix.

## 2 Materials and methods

### 2.1 Materials

1-palmitoyl-2oleoyl-sn-glycero-3-phosphocholine (POPC, 16:0–18:1), 1,2-dioleoyl-sn-glycero-3-phospho-(1′-rac-glycerol) (sodium salt) (DOPG, 18:1 (Δ9-Cis)) were purchased from Avanti Polar Lipids, USA. NaC hydrate (BioXtra, ≥99%), Tris base, (3,4′,5-Trihydroxy-trans-stilbene) (*t-*res), low molecular weight CH with 75–85% deacetylation, PGA with ≥85% (titration) from oranges, sodium tripolyphosphate pentabasic (TPP), Triton X-100, porcine pepsin (P7012), porcine pancreatin (P7545), bovine bile (B3883), Ammonium carbonate ((NH_4_)_2_CO_3_), Pefabloc SC, d-mannitol and D-(+)-glucose were purchased from Sigma Aldrich, UK. Chloroform, methanol, sodium chloride (NaCl), Ethylenediaminetetraacetic acid (EDTA), Calcium chloride dihydrate (CaCl_2_(H_2_O)_2_), sodium hydroxide (NaOH), Potassium chloride (KCl), Sodium bicarbonate (NaHCO_3_), and Magnesium chloride (MgCl_2_(H_2_O)_6_) were purchased from Merck, UK. Hydrochloric acid (HCl) and Potassium dihydrogen orthophosphate (KH_2_PO_4_) were purchased from J.T. Baker. Agar was purchased from VWR International. OM (Alpro, whole, 3.5% fat in **Table S1**) was purchased from a local grocery store (Tesco, Leeds, UK).

### 2.2 Preparation of *t*-res loaded liposomes, bilosomes, CH-coated, and PGA/CH-coated bilosomes

The *t-*res-loaded liposomes, bilosomes, CH-coated, and PGA/CH-coated bilosomes were prepared according to the thin-film rehydration method, followed by sonication with minor modifications (Coreta-Gomes et al., 2015). The minimum and maximum concentrations of the compounds used in the formulation were determined based on the literature (Imam et al., 2021; Waglewska et al., 2020; Wang et al., 2021). The molar ratios (mol%) of the samples are summarised in Table 1, and the corresponding actual molarities are listed in **Table S2.** For liposomes, POPC, DOPG, and *t-*res were dissolved in a mixture of chloroform and methanol (80:20, *v*/v), and solvents were evaporated using a Genevac evaporator (EZ-2 plus) (Fisher Scientific Ltd., Leicestershire, UK) (25 °C, Method: Very Low BP Mix) to obtain dried lipid films. The lipid films were hydrated with 25 mL Tris buffer (10 mM, pH 5.5) containing 150 mM NaCl and 1 mM EDTA and vortexed at room temperature (RT). The hydrated suspension underwent five freeze–thaw cycles (−196 °C/50 °C) and was sonicated using a QSonica Q125 Sonicator with a stepped microtip (Newtown, CT) for 10 min at 80% amp (1 s on/2 s off, 125 W max power). An ice-water bath was used to avoid damage from sample heating upon sonication. For bilosomes, a phase diagram was first constructed to identify NaC concentrations close to the critical micelle concentration based on hydrodynamic diameter (D_H_), polydispersity index (PDI), and zeta (ζ) potential (Garidel et al., 2007) (Data not given). Based on this analysis, different NaC molarities (5, 7.5, and 10 mM) were selected for bilosome preparation. The NaC was dissolved in a chloroform/methanol solution containing POPC, DOPC, and *t*-res, and the same procedure used for liposome preparation was followed. CH-coated bilosomes were prepared by adding bilosomes (pH 5.5) dropwise into a CH solution (3 mg/mL, pH 5.5) under stirring (500 rpm, RT). A 0.1% (*v*/v) TPP solution was added as a cross-linker and stirring continued overnight (Abbas et al., 2021; Ramezanzade et al., 2021). After adjusting the pH to 5.5, samples were sonicated in an ice-water bath (35% amplitude, 1 s on/2 s off, 10 min). NaC/CH ratios from 5.0 to 0.23 (*w*/w) were tested, with 0.5 (w/w) identified as optimal based on D_H_, PDI, and ζ potential (**Fig. S1).** For PGA/CH coating, CH-coated bilosomes were added dropwise into a PGA solution (3 mg/mL, pH 5.5) under stirring (500 rpm, RT) and left overnight (Lopes et al., 2021). The pH was readjusted to 5.5, and samples were sonicated in an ice-water bath (35% amp, 1 s on/2 s off, 3 min). CH/PGA ratios from 2 to 0.2 (w/w) were examined, with 0.4 (w/w) found optimal (**Fig. S2)**. All biopolymer-coated bilosomes were filtered through a 1.5 μm nylon filter and stored at 4 °C until further analysis. Each sample formulation was independently produced in triplicate to ensure reproducibility.

**Table 1:** Composition of *t*-res loaded liposomes, bilosomes, and CH- and PGA/CH-coated bilosomes.

System	Lipid (POPC:DOPG) (mol%)	Lipid: NaC (mol%)	Lipid: *t*-res (mol%)	NaC/CH (w/w)	CH/PGA (w/w)
L-5	75: 25	–	72.7: 27.3	–	–
B-5:5	75: 25	72.7: 27.3	72.7: 27.3	–	–
B-7.5:5	75: 25	64.0: 36.0	72.7: 27.3	–	–
B-10:5	75: 25	57.1: 42.9	72.7: 27.3	–	–
CH-B-5:5	75: 25	72.7: 27.3	72.7: 27.3	0.5	–
CH-B-7.5:5	75: 25	64.0: 36.0	72.7: 27.3	0.5	–
CH-B-10:5	75: 25	57.1: 42.9	72.7: 27.3	0.5	–
PGA/CH-B-5:5	75: 25	72.7: 27.3	72.7: 27.3	0.5	0.4
PGA/CH-B-7.5:5	75: 25	64.0: 36.0	72.7: 27.3	0.5	0.4
PGA/CH-B-10:5	75: 25	57.1: 42.9	72.7: 27.3	0.5	0.4

POPC: 2-oleoyl-1-palmitoyl-sn-glycero-3-phosphocholine; DOPG: 1,2-dioleoyl-sn-glycero-3-phospho-(1′-rac-glycerol) (sodium salt); NaC: Sodium cholate; *t-*res: trans-resveratrol; CH: Chitosan; PGA: Polygalacturonic acid.

### 2.3 Characterization of *t*-res loaded delivery systems

#### 2.3.1 Hydrodynamic diameter, polydispersity index, and zeta potential

The D_H_ and PDI of samples were determined by dynamic light scattering (DLS) using a Zetasizer Nano ZS series (Malvern Instruments, UK) at 25 °C and 173° backscatter. Liposome refractive index and absorption were 1.45 and 0.001, respectively. The ζ potential was measured at 90°, and all measurements were performed in triplicate. Intestinal phase digesta samples were centrifuged at 280 ×*g* for 5 min (Thermo Scientific Fresco 21), and the supernatant was used for DLS.

#### 2.3.2 Quantification of *t*-res

The amount of free *t-*res in the samples was quantified at 310 nm using the high-performance liquid chromatography-diode array detector (HPLC-DAD) (Shimadzu, Japan) system controlled by LabSolutions software (version 5.97) (Ares et al., 2015). Separation was performed on an Ascentis® Express C18 column (2.7 μm, 15 cm × 4.6 mm) protected by a Phenomenex (AJ0–4287) C18 guard cartridge (4 × 3.0 mm) at 30 °C*. mobile* phases were 1% (*v*/v) formic acid in water and acetonitrile, with a flow rate of 0.8 mL/min and a 33 min. Gradient using solvent (A) 1% formic acid in water and (B) acetonitrile. A 50 μL filtrate was injected under the following gradient: (i) 0–21 min, 71:29 v/v (A:B); (ii) 24–27 min, 0:100 v/v (A:B); (iii) 30–33 min, 71:29 v/v (A:B). Eluted *t*-res was detected at 310 nm, and all measurements were performed in triplicate (Ares et al., 2015).

#### 2.3.3 Encapsulation efficiency and loading capacity

Free *t*-res was separated by centrifuging 0.5 mL liposome and bilosome samples at 108800 x g for 60 min (Beckman Coulter Avanti J-30I) and 1.5 mL biopolymer-coated bilosomes at 21000 x g for 60 min, both at 4 °C (Isailović et al., 2013; Wang et al., 2025). To determine the EE% of bilosomes, the pellets were collected, whereas for biopolymer-coated bilosomes, the supernatants were collected. The collected fractions were mixed with methanol and dried using a Genevac EZ-2 Plus evaporator (25 °C, Method: Aqueous). The dried samples were dissolved in methanol, filtered (0.20 μm, PTFE), and analysed by HPLC-DAD to determine free and encapsulated *t*-res (Ares et al., 2015). EE% and LC% were calculated using eqs. (1), (2) respectively. Measurements were performed in triplicate.

**(1)** $$\mathrm{EE}\%=\frac{\text{total}\;t\mathrm{res}\;\left(\mathrm{mg}\right)-\text{free}\;t\mathrm{res}\;\left(\mathrm{mg}\right)}{\text{total}\;t\mathrm{res}\;\left(\mathrm{mg}\right)}x100$$

**(2)** $$\mathrm{LC}\%=\frac{\text{total}\;t\mathrm{res}\;\left(\mathrm{mg}\right)-\text{free}\;t\mathrm{res}\;\left(\mathrm{mg}\right)}{\text{total}\;t\mathrm{res}\;\left(\mathrm{mg}\right)+\text{lipids}\;\left(\mathrm{mg}\right)+\mathrm{NaC}\;\left(\mathrm{mg}\right)}x100$$

### 2.4 Static *in vitro* gastrointestinal digestion

The Infogest *in vitro* digestion protocol was followed to assess the fate of empty and *t-*res-loaded liposomes, bilosomes, and CH-coated and PGA/CH-coated bilosomes during digestion (Brodkorb et al., 2019). The effect of including an example food matrix on the behaviour of the samples during digestion was investigated with the addition of OM (pH 7.64 and 3.5% fat) (1:1 *v*/v) to mimic food fortified with *t*-res in different delivery vehicles. Simulated salivary fluid (SSF, pH 7), SGF (pH 3), and SIF (pH 7) were prepared according to **Table S3,** and enzyme activity assays were performed according to supplementary data 1 and 2 of INFOGEST protocol (Brodkorb et al., 2019). All electrolyte stock solutions of digestion fluids were warmed to 37 °C, and enzymes and bile solutions were prepared immediately before digestion steps. Samples were collected at the beginning and end of the oral phase and after 5 and 120 min for the gastric and intestinal phases. ***Oral phase:*** A 10 mL sample was mixed with SSF (5:4, v/v) and 0.05 mL 300 mM CaCl₂·(H₂O)₂ (final concentration: 1.5 mM in SSF) was added. The CaCl_2_.(H_2_O)_2_ was added immediately before the digestion experiment to minimize precipitation during incubation. Deionised water was added to obtain a 1:1 (*v*/v) sample-to-SSF ratio, and the mixture was incubated at 37 °C, 100 rpm for 2 min (SciQuip Incu-Shake MAXI). ***Gastric phase:*** The oral bolus was mixed with pre-warmed SGF (5:4, v/v), followed by porcine pepsin (2000 U/mL) and 0.01 mL 300 mM CaCl₂·(H₂O)₂ (final concentration: 0.15 mM in SGF), the latter added immediately before digestion. The pH was adjusted to 3 using 1 M HCl, and deionised water was added to achieve a 1:1 (v/v) oral bolus-to-SGF ratio. The mixture was incubated at 37 °C, 100 rpm for 2 h. In collected samples, pepsin activity was stopped by raising the pH to 7 with 1 M NaHCO₃. ***Intestinal phase:*** Gastric chyme was mixed with pre-warmed SIF (2.35:1, v/v), followed by bile (10 mM) and porcine pancreatin (trypsin activity: 100 U/mL). 0.08 mL 300 mM CaCl₂·(H₂O)₂ (final concentration: 0.6 mM in SIF) was added immediately before digestion, and the pH was adjusted to 7 using 1 M NaOH. Deionised water was added to achieve a 1:1 (v/v) gastric chyme-to-SIF ratio. The mixture was incubated at 37 °C, 100 rpm for 2 h. In collected samples protease activity was stopped with the addition of Pefabloc (final concentration: 5 mM). All samples taken from the static *in vitro* digestion experiments were flash frozen in liquid nitrogen and then stored at −80 °C until further analysis. In addition, changes in samples during simulated digestion were evaluated by D_H_, PDI, and ζ potential. All measurements were performed in triplicate. In addition, a blank *in vitro* digestion experiment without samples was conducted to evaluate the effects of pH changes, enzyme activity, and buffer addition on hydrodynamic diameter (D_H_), polydispersity index (PDI), and zeta (ζ) potential and was used as a control for comparison with the samples.

### 2.5 Bioaccessibility determination

The amount of *t-*res released at the end of *in vitro* digestion was determined as described previously with slight modifications (Niaz et al., 2021). The raw digesta from the intestinal phase was collected and centrifuged at108800 x g for 60 min at 4 °C, which gave a sediment at the bottom and a clear supernatant at the top. The supernatant micellar phase was collected (Tikekar et al., 2013)and dried using a Genevac (EZ-2 plus) evaporator (25 °C, Method: Aqueous). The dried samples were dissolved in methanol and filtered (0.20 μm, PTFE) for quantification of *t-*res using HPLC-DAD. The bioaccessibility of *t-*res was calculated using eq. 3. Measurements were performed in triplicate. Free *t*-res and bilosomes were used as controls to evaluate the effects of encapsulation and biopolymer coating on the bioaccessibility of *t*-res, respectively.

**(3)** $$\text{Bioaccessibility}\;\left(\%\right)=\frac{t\mathrm{res}\;\text{in}\;\mathrm{raw}\;\text{digesta}\;\left(\mathrm{mg}\right)}{\text{total}\;t\mathrm{res}\;\left(\mathrm{mg}\right)}x100$$

### 2.6 Determination of *t*-res murine intestinal absorption

To assess the intestinal absorption of free *t-*res and *t-*res loaded in liposomes and bilosomes, the Ussing chamber method (Mackie et al., 2019; Niaz & Mackie, 2024) was used.

***Tissue preparation and mounting:*** Intestines from C57BL/6 mice were collected immediately after sacrifice, flushed twice with ice-cold Krebs–Ringer bicarbonate (KRB) solution, and placed into an ice-cold 10 mM glucose solution. The intestine was longitudinally opened, and the serosa and muscularis layers, which are non-absorptive and not directly involved in nutrient or compound transport across the intestinal mucosa, were carefully removed (Noorman et al., 2024). The prepared duodenal tissue was mounted on a 0.25 cm^2^ tissue insert (P2404, Physiologic Instruments, San Diego, USA) and placed into a stabilized Ussing chamber system (EM-CSYS-4). ***Absorption experiments:*** After mounting, intestinal tissue was inserted into the Ussing chamber. The apical compartment was filled with 1 mL Ringer's solution (120 mM NaCl, 3 mM KCl, 0.5 mM MgCl₂.6H₂O, 1.25 mM CaCl₂.2H₂O, 23 mM NaHCO₃) containing 10 mM mannitol, and the basolateral side with 1 mL Ringer's solution containing 10 mM glucose to maintain osmotic balance. The tissue was equilibrated for 30 min before the sample addition into the apical chamber. The chamber temperature was maintained at 37 °C using a circulating water bath, and oxygenation was provided *via* a carbogen gas supply (5% CO₂/95% O₂). After equilibration, 0.5 mL of apical buffer was replaced with 0.5 mL of digesta. Samples (0.3 mL) were collected from both apical and basolateral sides at 60 and 120 min and replaced with fresh buffer to maintain a 1 mL volume. The *t*-res content was quantified as described in Section 2.3.2. At the end of the experiment (120 min), mounted tissues were collected to determine *t*-res accumulation. Tissue viability was continuously assessed *via* transepithelial electrical resistance (TEER) (**Fig. S3**), calculated using Ohm's law (Eqs. (4), (5)), and only tissues with TEER >20 Ω.cm^2^ were included in the analysis (Guan et al., 2022). Free *t*-res and liposomes collected at the end of the intestinal phase were used as controls to evaluate the effects of encapsulation and NaC incorporation respectively, on the absorption of *t*-res. (PD: Transepithelial potential difference; Isc: Short circuit current).

**(4)** $$\text{Resistan}\mathrm{ce}=\mathrm{PD}\;\left(\mathrm{mV}\right)/\mathrm{Isc}\;\left(\mathrm{mA}\right)$$

**(5)** $$\text{TEER}\;\left(\Omega .cm^{2}\right)=\text{Resistan}\mathrm{ce}\;\left(\Omega \right)\;x\;\text{area}\;\left(cm^{2}\right)$$

### 2.7 Statistical analysis

All experiments were performed in independent replicates, and the results are expressed as mean ± standard deviation (SD). Statistical analyses were conducted using Minitab® software (version 20.4; Minitab Inc., USA). Differences among experimental groups were evaluated using one-way analysis of variance (ANOVA). When a significant effect was detected, Tukey's multiple comparison *post hoc* test was applied to identify statistically significant differences between groups and to account for multiple comparisons. Prior to ANOVA, the assumptions of normality and homogeneity of variances were assessed using standard diagnostic procedures. A *p*-value <0.05 was considered statistically significant (Agbangba et al., 2024).

## 3 Results and discussion

### 3.1 The initial properties and *in vitro* digestion characteristics of liposomes and bilosomes digested without/with OM

Changes in free *t*-res after simulated digestion were evaluated by measurement of D_H_, PDI, and ζ potential **(Fig. S4A, Table S4)**. The particle characterization of the *t*-res suspension (crystals in water) was not feasible due to the difficulty of obtaining representative samples. When free *t*-res was exposed to simulated salivary fluid (SSF, pH 7), the D_H_ was ∼1200 nm with a PDI of ∼0.800, reflecting poor solubility and the presence of suspended crystals, while the ζ potential was −3 mV. In the gastric phase, both D_H_ and PDI decreased significantly to ∼250 nm and ∼ 0.300, respectively (*p* < 0.05). Upon transition to the intestinal phase, the D_H_ of the gastric chyme of free *t*-res increased to ∼350 nm, whereas the magnitude of ζ potential increased from −5 mV to around 12 mV (p < 0.05), changes likely associated with the formation of negatively charged bile salt–containing micelles. When free *t*-res was mixed with OM, the resulting t-res–OM mixture (pH 7.35) exhibited a D_H_ of 380 ± 3.4 nm, a PDI of ∼0.300, and a ζ potential of −22.6 ± 0.899 mV, values comparable to those of OM alone, indicating that the mixture was predominantly governed by the physical properties of OM. After exposure to SSF, the properties of the t-res–OM mix remained unchanged (*p* > 0.05). However, upon gastric digestion, the D_H_ increased from ∼420 nm (PDI: 0.093) to ∼800 nm (PDI: 0.800). As digestion progressed, D_H_, PDI, and the magnitude of the ζ potential increased (p < 0.05). In the subsequent intestinal phase, the D_H_ and PDI of the gastric chyme decreased to ∼550 nm and 0.220, respectively, within the first 5 min and remained stable thereafter. Concurrently, the magnitude of ζ potential initially decreased to about −13 mV but gradually increased to about −20 mV by the end of intestinal digestion (p < 0.05), which may be attributed to the release of negatively charged fatty acids during lipid hydrolysis.

***Liposomes and bilosomes:*** The initial D_H_, PDI, and ζ potential values of *t*-res-loaded liposomes and bilosomes before and after mixing with OM, as well as EE% and LC%, are given in Table 2**.** The initial D_H_ of *t*-res-loaded liposomes was 263.0 ± 2.1 nm with a ζ potential of −40.9 ± 4.0 mV. A progressive increase in D_H_ was observed from B-5:5 (391.0 ± 7.5 nm) to B-7.5:5, which reached the highest value (428.8 ± 2.7 nm). However, further increasing NaC to 10 mM (B-10:5) resulted in a significant size reduction (370.4 ± 7.6 nm), although the value remained higher than that of the control liposomes. The addition of 5 mM NaC significantly increased the magnitude of ζ potential (−46.4 ± 1.6 mV) (*p* < 0.05), although further increases in NaC concentration did not cause significant changes. The EE% of liposomes were 25.3 ± 0.2% with an LC% of 2.5%, while bilosomes showed a significantly higher EE% (41.4 ± 0.4% to 45.4 ± 3.5%) and LC% (∼3.5%) (p < 0.05). The presence of NaC in the lipid bilayers enhanced the EE%, probably because the bile salts altered the packing of the molecules within the hydrophobic domains of the lipid bilayers (Cheng et al., 2019). Previous studies reported an LC% of ∼3.1% for resveratrol-loaded liposomes (Soo et al., 2016), while curcumin-loaded liposomes modified with rhamnolipids achieved EE% of over 90% and LC% above 3.5% (Chen et al., 2022).

**Table 2:** Properties of *t*-res loaded liposomes and bilosomes, without/with OM at pH 5.5.

	System	D_H_ (nm)	PDI	ζ potential (mV)	EE%	LC%
Without OM	L-5	263.0 ± 2.1^d^	0.225 ± 0.040^b^	−40.9 ± 4.0^c^	25.3 ± 0.2^b^	2.5 ± 0.1^b^
	B-5:5	391.0 ± 7.5^bc^	0.259 ± 0.040^b^	−46.4 ± 1.6^d^	41.4 ± 0.4^a^	3.5 ± 0.1^a^
	B-7.5:5	428.8 ± 2.7^a^	0.254 ± 0.010^b^	−46.4 ± 1.0^d^	42.8 ± 0.7^a^	3.3 ± 0.1^a^
	B-10:5	370.4 ± 7.6^c^	0.264 ± 0.023^b^	−47.9 ± 2.9^d^	45.4 ± 3.5^a^	3.3 ± 0.3^a^
With OM	L-5	416.4 ± 2.6^ab^	0.426 ± 0.007^a^	−17.2 ± 0.4^a^	–	–
	B-5:5	419.1 ± 10.5^a^	0.429 ± 0.018^a^	−20.8 ± 0.7^b^	–	–
	B-7.5:5	413.9 ± 7.2^ab^	0.419 ± 0.010^a^	−21.0 ± 0.4^b^	–	–
	B-10:5	408.7 ± 12.0^ab^	0.402 ± 0.004^a^	−22.4 ± 0.1^b^	–	–

OM: Oat milk; D_H_: Hydrodynamic diameter; PDI: Polydispersity index; ζ: Zeta; EE%: Encapsulation efficiency; LC%: Loading capacity. Data are presented as the mean ± standard deviation of three independent measurements (*n* = 3). Different superscript letters indicate statistically significant differences between samples within the same column (*p* < 0.05).

**Fig. 1:**
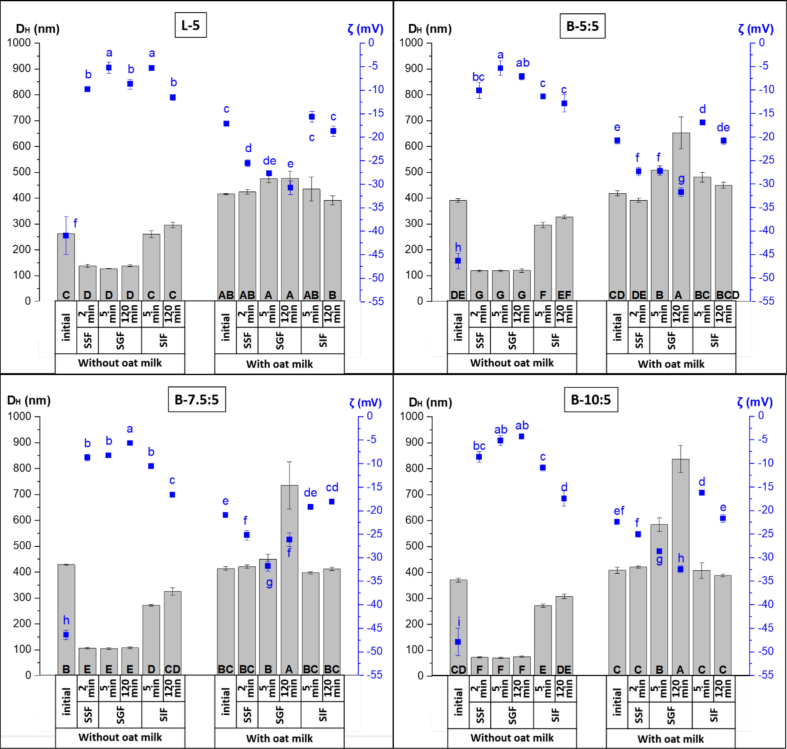
Effect of exposure to different phases of the static *in vitro* gastrointestinal digestion model on the hydrodynamic diameter (D_H_, nm, ) and zeta (ζ) potential (mV, ■) of trans-resveratrol (*t*-res) loaded liposomes and bilosomes without and with oat milk. Error bars for D_H_ (Ɪ) and ζ potential (Ɪ) indicate the standard deviation (SD) obtained from three independent measurements (mean ± SD, *n* = 3). Capital letters and lowercase letters indicate the significant differences in D_H_ and ζ potential of the samples, respectively (*p* < 0.05). (SSF: simulated salivary fluid, SGF: simulated gastric fluid, SIF: simulated intestinal fluid).

Changes in *t*-res-loaded liposomes and bilosomes **(**Fig. 1**, Table S4)**, after simulated digestion, were evaluated by measurement of D_H_, PDI, and ζ potential. Upon exposure to SSF in the oral phase, both the magnitude of ζ potential and D_H_ of the liposomes and bilosomes decreased to around −10 mV and ∼ 100 nm, respectively. The charge decrease was likely due to the ionic screening effect of salts in SSF and the change in pH (Singh & Sarkar, 2011). The reduction in D_H_ may be attributed to the pH shift from 5.5 to 7.0, which likely induced vesicle restructuring and interactions with cations (K^+^, Na^+^, Ca^2+^) in SSF, promoting osmotic dehydration of the bilayer and consequent shrinkage. Furthermore, these ions may adsorb onto the bilayers, altering membrane curvature and thereby affecting vesicle shape and lamellarity (Hupfeld et al., 2010). In the gastric phase, liposomes and bilosomes showed minimal changes upon exposure to simulated gastric fluid (SGF, pH 3), indicating good stability under gastric conditions. These findings are consistent with those of Hui and Huang, who reported no significant change in D_H_ but slight changes in the ζ potential of liposomes during the gastric phase (Hui & Huang, 2021). Upon exposure to the intestinal phase, the D_H_ of liposomes and bilosomes of gastric chyme increased significantly (up to ∼300 nm) after exposure to SIF (pH 7) (*p* < 0.05) and remained stable until the end of the intestinal phase. Firstly, this increase was attributed to the action of porcine pancreatin and bile salts (control SIF: D_H_ ∼ 220 nm, PDI ∼0.270). As previously noted, bile salts can induce membrane curvature in phospholipid bilayers, leading to a variety of lipid structures, including ellipsoid shapes, rod-like formations, smaller vesicles, and micelles (Garidel et al., 2007). Since the medium contains multiple size distributions, DLS calculates the D_H_ assuming spherical particle, but the presence of diverse morphologies can influence the measured D_H_ (Kiselev et al., 2013). Additionally, the magnitude of the ζ potential of both carriers increased significantly from about −5 mV to −15 mV (*p* < 0.05), consistent with previous findings for torularhodin- and epigallocatechin-loaded liposomes and bilosomes (Liu, Guo et al., 2022; Wang et al., 2021). This shift is likely attributed to the presence of negatively charged bile salts, free fatty acids, and other anionic species in the lipid structures formed during digestion (Zhang et al., 2015). By the end of the intestinal phase, D_H_ and PDI did not differ significantly between liposomes and bilosomes.

***Liposomes and bilosomes co-digested with OM:*** The initial D_H_, PDI, and ζ potential of the OM (pH 7.64) were determined as 390.3 ± 11.2 nm, 0.398 ± 0.008, and − 22.5 ± 0.7 mV, respectively**.** After mixing liposomes and bilosomes (pH 5.5) with OM (Table 2), the D_H_ values were considerably larger for all formulations (408.7 ± 12.0 to 419.1 ± 10.5 nm) compared to their counterparts without OM (p < 0.05). PDI values also increased to ∼0.40–0.43, indicating reduced homogeneity. The final pH of the delivery system–OM mixtures was ∼7.35. The ζ potential magnitude decreased from about −45 mV to −20 mV upon mixing (p < 0.05), suggesting that the dilution effect, the change in pH, and interactions with OM may mask surface charges or induce structural rearrangements at the vesicle interface (Zhang et al., 2022). The similarity in properties between OM and the delivery system-OM mixtures, along with the absence of significant differences among NaC concentrations, indicates that OM dominated the measured characteristics under these conditions.

Changes in *t*-res-loaded liposomes and bilosomes **(**Fig. 1**, Table S4)**, after simulated digestion with OM, were evaluated by measurement of D_H_, PDI, and ζ potential. For the liposome-OM mix and the bilosome-OM mix, no significant changes in D_H_ were observed after SSF exposure compared to their initial properties. However, in contrast to the liposomes (L-5) and bilosomes (B-5:5, B-7.5:5, and B-10:5), the magnitude of the ζ potential of both the liposome-OM and bilosome-OM mix increased and was around −25 mV after exposure to the SSF, and the PDI significantly decreased to ∼0.150 (*p* < 0.05). The increased magnitude of the ζ potential might be due to the deprotonation of carboxyl groups of proteins OM, contributing to the negative surface charge. Upon exposure of the oral bolus of the liposome-OM mix and the bilosome-OM mix to SGF, a slight increase in D_H_ was observed. This increase may be attributed to the destabilisation and coalescence of oil droplets and protein aggregation caused by the enzymatic digestion of OM proteins by pepsin (Mackie & Macierzanka, 2010; Zheng et al., 2021). Compared to the liposome–OM mix, the D_H_ of the bilosome–OM mix was larger at the end of the gastric phase, and the D_H_ further increased with higher concentrations of NaC in the bilosomes. This change in D_H_ may be attributed to protein–NaC interactions. At the low pH of the gastric phase (pH ∼3), proteins generally carry a net positive charge due to the protonation of amino groups and other basic functional groups (Van der Sman et al., 2020; Zhang et al., 2022). Although NaC is only partly deprotonated, it still provides a slight negative charge. This allows NaC to be electrostatically attracted to positively charged protein regions, promoting binding or complex formation. Such interactions can induce aggregation and conformational changes in proteins, which may contribute to the observed increase in D_H_ (Bellesi & Pilosof, 2021; De et al., 2007). Moreno et al. suggested that proteins in their molten globule state, induced by acidic pH, can associate with phospholipid bilayers (Moreno et al., 2005). Additionally, secreted phospholipids may displace adsorbed proteins on the surface of oil droplets, contributing to structural changes (Dupont et al., 2010; Wang et al., 2020). A similar significant increase in D_H_ from the oral to the gastric phase has also been reported for soymilk (Lv et al., 2024; Zheng et al., 2019). The magnitude of the ζ potential in the oral bolus of the delivery systems-OM mixture also increased during the gastric phase (*p* < 0.05). These findings agree with previous studies on the digestion behaviour of plant-based milks such as oat, almond, coconut, and cashew (Zheng et al., 2021). Upon exposure of the gastric chyme of the liposome-OM mixture to SIF, no significant changes in D_H_, PDI, or ζ potential were observed. In contrast, the bilosome–OM mixture showed a decrease in D_H_ to ∼450 nm, possibly due to the hydrolysis or disassociation of large bilosome–OM aggregates formed in the gastric phase into smaller, more dispersed structures (Mackie & Macierzanka, 2010). The ζ potential was negative due to the negatively charged bile salts, OM proteins, and free fatty acids under SIF conditions. A decrease in ζ potential magnitude from approximately −30 mV to −15 mV after 5 min of intestinal digestion (*p* < 0.05) was likely due to the buffering capacity of OM and the ionic strength of the SIF, which can mask surface charges even though solubilization and structural reorganisations are still occurring (Lv et al., 2024; Waraho et al., 2011).

### 3.2 Bioaccessibility of *t*-res in liposomes and bilosomes digested without/with OM

**Fig. 2:**
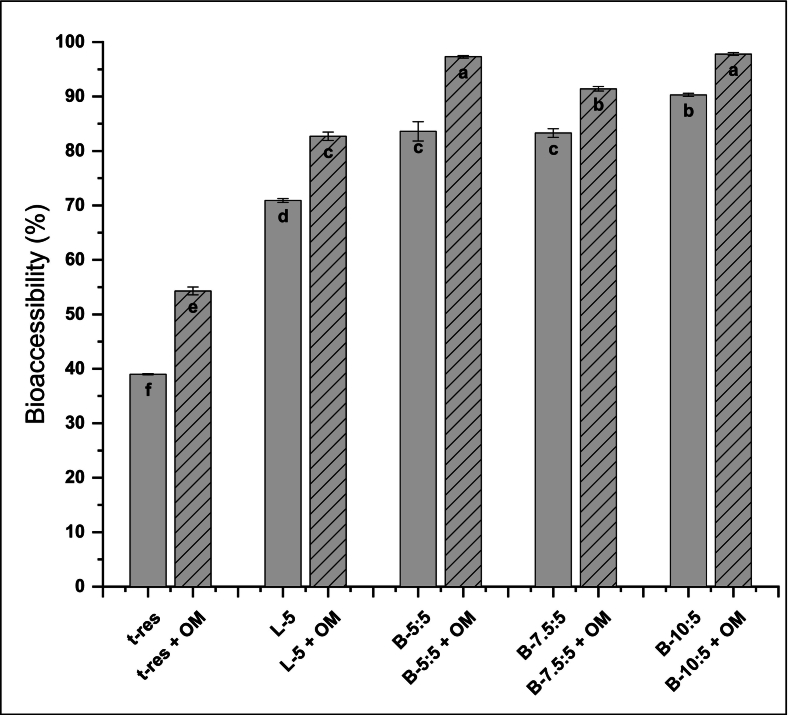
Concentration and bioaccessibility (%) of trans*-*resveratrol (*t*-res) in liposomes and bilosomes digested without () and with oat milk (). Error bars for bioaccessibility (Ɪ) indicate the standard deviation (SD) obtained from three independent measurements (mean ± SD, n = 3). Letters indicate the significant differences in bioaccessibility of *t-*res from the samples (p < 0.05).

After *in vitro* digestion of the liposomes and bilosomes, the amount of *t*-res present in the mixed micelle phase was quantified (Fig. 2). For comparison, *t*-res suspended in Tris buffer was used as a control. In addition, the influence of OM on the bioaccessibility of *t*-res was evaluated.

***Liposomes and bilosomes:*** The bioaccessibility of free *t*-res was 39.0 ± 0.1%. Encapsulation into liposomes significantly enhanced this value by 1.82-fold (*p* < 0.05), consistent with previous reports. For instance, Toro-Uribe et al. reported 2.3-fold and 2.2-fold increases in the bioaccessibility of catechin and epicatechin, respectively, upon liposomal encapsulation (Toro-Uribe et al., 2018). The improved bioaccessibility can be attributed to the encapsulation of bioactive compounds within lipid bilayers, which enhances their solubility and stability while reducing degradation by controlling their release (McClements, 2020). Further enhancement was achieved with the incorporation of NaC into the system. The bioaccessibility of *t*-res increased to a range of 2.14–2.32-fold, specifically reaching up to 90.3 ± 0.3% in B-10:5 (p < 0.05). This trend aligns with the findings of Jeong et al., who reported increased bioaccessibility of avenanthramide by ∼1.20-fold with liposomes and ∼ 1.39-fold with bilosomes (Jeong et al., 2025) and increased bioaccessibility of epigallocatechin gallate (EGCG) from 3.1 ± 0.4% (free EGCG) to 24.0 ± 3.9%, 55.7 ± 6.9%, and 71.7 ± 4.1% when encapsulated into liposomes, niosomes, and bilosomes, respectively (Wang et al., 2021).

The enhanced performance of bilosomes can be attributed to their increased amphiphilicity and bilayer fluidity due to NaC, which promotes bilayer disruption and the formation of diverse lipid nanostructures (*e.g.*, ellipsoids, rods, vesicles, micelles), thereby improving the LC% and ultimately influencing the amount of bioaccessible *t*-res (Lichtenberg et al., 2013; McClements, 2013). Consistent with this, the LC% of liposomes increased from 2.5% to 3.3–3.5% with the addition of NaC to the formulations (p < 0.05) (Table 2). During digestion, bile salts facilitate the formation of mixed micelles with phospholipids and digestion products, thereby improving *t*-res solubilization in the intestinal environment (Macierzanka et al., 2019). Additionally, the presence of bile salts may enhance the interaction of bilosomes with intestinal membranes by loosening tight junctions and improving membrane permeability, which collectively contribute to increased *t*-res absorption (Azman et al., 2022; Moghimipour et al., 2015).

***Liposomes and bilosomes co-digested with OM:*** Co-digestion with OM significantly enhanced the bioaccessibility of both free *t*-res and *t*-res encapsulated in liposomes or bilosomes (p < 0.05). For liposomes, bioaccessibility increased by 1.17-fold, whereas for bilosomes it ranged from 1.08-fold to 1.16-fold compared to their respective counterparts (p < 0.05), in agreement with previous reports. Co-digestion with whole milk increased the bioaccessibility of β-carotene from microparticles by approximately 1.5-fold and 2-fold compared to semi-skimmed and skimmed milk, respectively (Cabezas-Terán et al., 2025). Zheng et al. reported similar improvements in the bioaccessibility of hydrophobic bioactives; for instance, curcumin bioaccessibility was ∼60% when digested with plant-based milks such as almond, cashew, coconut, and OM (Zheng et al., 2021). The enhanced bioaccessibility of *t*-res in liposomes and bilosomes during co-digestion may be attributed to the triacylglycerol-rich core of the oil droplets in OM. During digestion, these triacylglycerols are hydrolysed by pancreatic lipases, producing mixed micelles that can solubilize *t*-res in the intestinal phase (Zheng et al., 2021). Niaz et al. demonstrated that the bioaccessibility of carvacrol loaded into chitosan/bovine serum albumin core–shell nanocarriers increased 1.14-fold when co-digested with skimmed milk (Niaz et al., 2021). These findings suggest that not only does the lipid fraction in the milk facilitate the micellar phase, but also other components, like metabolites from protein digestion or molecular interactions that protect the bioactive from degradation, may contribute to enhanced bioaccessibility (Chen et al., 2018; Niaz et al., 2021).

### 3.3 *Ex vivo* absorption of *t*-res through murine intestinal tissue in Ussing chambers

**Fig. 3:**
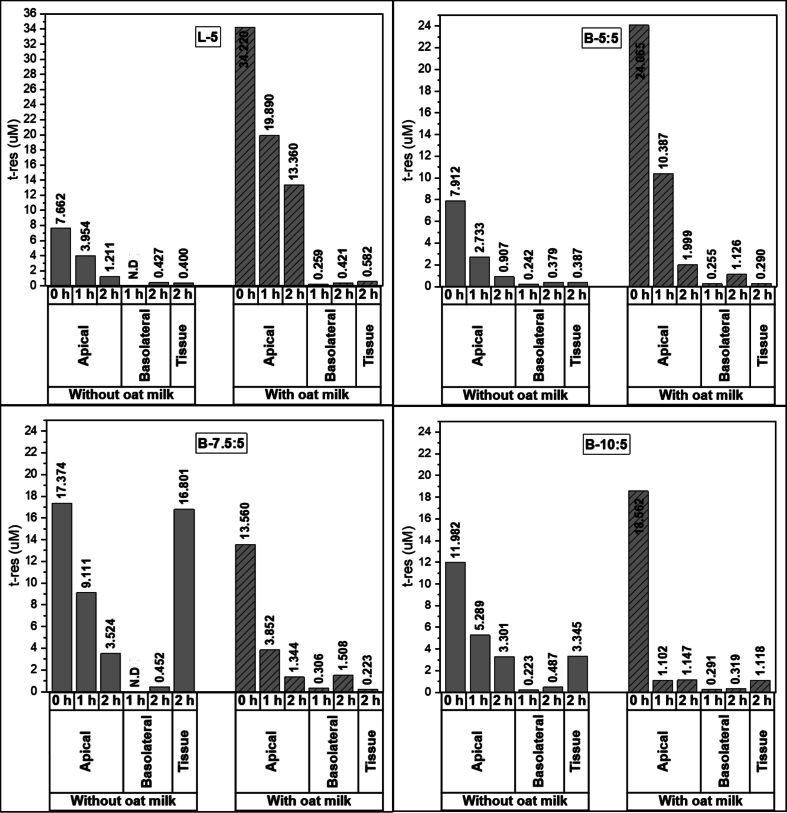
Concentration changes of trans-resveratrol (*t*-res) () and *t*-res digested with oat milk () in the apical and basolateral chambers after exposure to the murine intestinal mucosa, and the concentration of accumulated *t-*res in the tissue for liposomes and bilosomes.

The absorption of free *t*-res, as well as *t*-res encapsulated in liposomes and bilosomes, digested in the absence or presence of OM, was evaluated using murine intestinal tissue mounted in Ussing chambers (Fig. 3**-S3B).** Samples of the intestinal digesta were placed in the apical compartment of the chambers, with initial *t*-res concentrations varying due to differences in bioaccessibility, as shown in Fig. 3**.** Notably, *t*-res is known to degrade under environmental conditions such as exposure to air, light, and temperature fluctuations, which may have occurred during sample preparation, digestion, and absorption experiments (Jurczyk et al., 2022). A progressive decrease in *t-*res concentration within the apical chamber was observed over 120 min for all samples, regardless of whether they were digested with or without OM. Throughout the experiment, an increase in *t-*res concentration was observed in the basolateral compartment. However, the *t*-res concentration at the 1-h time point remained below the detection limit for liposomes and B-7.5:5 samples**.** At the 2-h time point, the *t*-res concentration in the basolateral compartment ranged from 0.379 μM to 0.487 μM for free *t*-res, liposomes, and bilosomes. While the amount of *t*-res that accumulated in the intestinal tissue was 0.400 μM for liposomes. Significantly higher levels were observed for bilosomes, with 16.801 μM for B-7.5:5 and 3.345 μM for B-10:5. These findings are consistent with previous research.

For example, Guan et al. reported higher oral absorption of cyclosporine A with soybean phosphatidylcholine/sodium deoxycholate liposomes (120.3%) compared to conventional soybean phosphatidylcholine/cholesterol liposomes (98.6%) (Guan et al., 2011). Consistently, Jeong et al. found that bilosomes achieved greater cellular uptake in Caco-2 cells than liposomes (Jeong et al., 2025). As discussed in the bioaccessibility section, differences in the accumulation of *t*-res within intestinal tissue are likely associated with the varying concentrations of NaC incorporated into the bilayer structure. The physicochemical characteristics (such as size, morphology, and surface charge) of these lipid-based nanocarriers influence their absorption behaviour (Lichtenberg et al., 2013; McClements, 2013). In addition, lower levels of *t*-res accumulation in the intestinal tissue were recorded for samples co-digested with OM, compared to those without OM. Specifically, the tissue accumulation of free *t*-res was 1.661 μM in the absence of OM and dropped to 0.417 μM when co-digested with OM **(Fig. S4B)**. As demonstrated in the *in vitro* digestion results **(**Fig. 2**)**, co-digestion with OM resulted in a higher bioaccessibility of *t*-res compared to the free system. Despite this increased bioaccessible fraction, *t*-res accumulation in intestinal tissue during the Ussing chamber experiments was lower when OM was present. This inverse relationship indicates that food matrix interactions, rather than differences in bioaccessible concentration, governed intestinal uptake. Mechanistically, *t*-res–loaded bilosomes undergo digestion similarly to dietary lipids, with encapsulated compounds incorporated into mixed micelles that can cross the intestinal epithelium *via* portal or lymphatic transport routes (Roger et al., 2010; Wang & Luo, 2019). Although resveratrol exhibits high membrane permeability due to its hydrophobicity (LogP ≈ 3.1), its absorption is modulated by mucus diffusion and epithelial transport barriers (McClements, 2015). OM contains several matrix components, including β-glucans, plant proteins, unsaturated lipids, phospholipids, and phytosterols, which can collectively modulate the absorption of hydrophobic compounds. β-glucans may increase digesta viscosity, thereby slowing mixed micelle diffusion and potentially entrapping bioactive compounds (Mackie et al., 2016; Niaz & Mackie, 2024). Moreover, soluble β-glucan fractions may inhibit lipolysis, delaying micelle formation and reducing the availability of *t*-res for epithelial uptake (Grundy et al., 2018). In addition, oat proteins such as globulins can interact with polyphenols through hydrophobic interactions and hydrogen bonding, forming protein–polyphenol complexes that may further alter absorption kinetics (Sęczyk et al., 2019; Wang et al., 2022). As a result, bilosome formulations (particularly B-7.5:5 and B-10:5) demonstrated significantly enhanced *t*-res absorption compared to both free *t*-res and liposomal formulations.

### 3.4 The initial properties, *in vitro* digestion characteristics, and bioaccessibility of CH-coated and PGA/CH-coated bilosomes, digested without/with OM

**Fig. 4:**
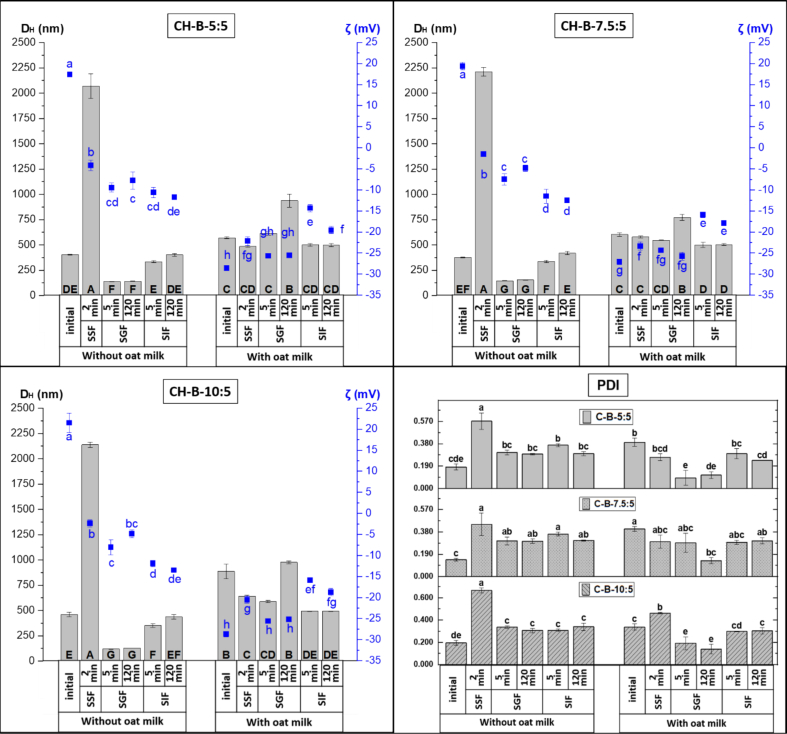
Effect of exposure to different phases of the static *in vitro* gastrointestinal digestion model on the hydrodynamic diameter (D_H_, nm, ), zeta (ζ) potential (mV, ■) and polydispersity index (PDI) of trans-resveratrol (*t*-res) loaded chitosan (CH)-coated bilosomes without and with oat milk. Error bars for D_H_, and PDI (Ɪ) and ζ potential (Ɪ) indicate the standard deviation obtained from three independent measurements (mean ± SD, n = 3). Capital letters, blue lowercase letters, and black lowercase letters indicate significant differences in D_H_, ζ potential and PDI of the samples, respectively (p < 0.05). (SSF: simulated salivary fluid, SGF: simulated gastric fluid, SIF: simulated intestinal fluid). (For interpretation of the references to colour in this figure legend, the reader is referred to the web version of this article.)

**Fig. 5:**
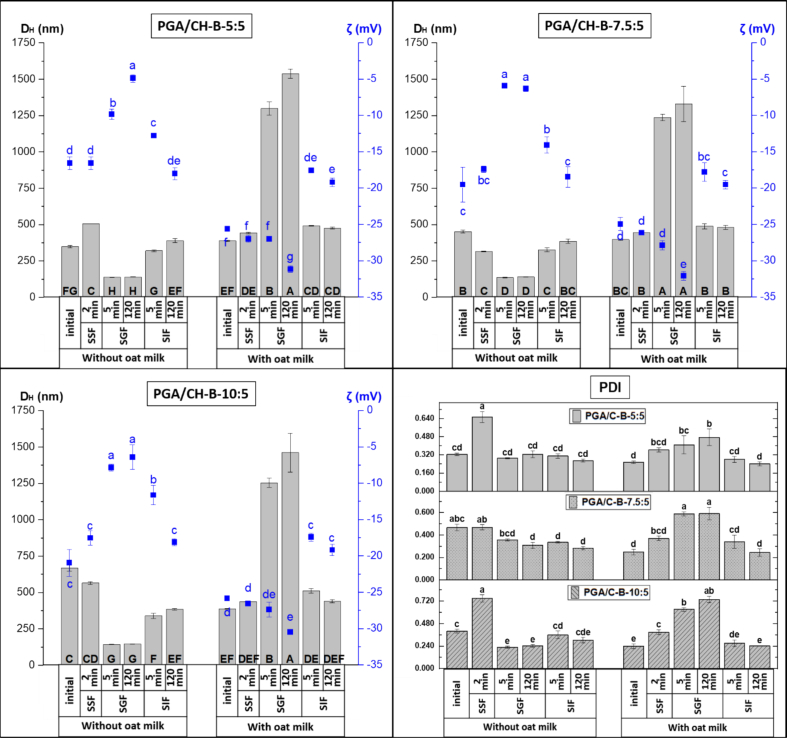
Effect of exposure to different phases of the static *in vitro* gastrointestinal digestion model on the hydrodynamic diameter (D_H_, nm, ), zeta (ζ) potential (mV, ■) and polydispersity index (PDI) of trans-resveratrol (*t*-res) loaded polygalacturonic acid (PGA)/ chitosan (CH)-coated bilosomes without and with oat milk. Error bars for D_H_, and PDI (Ɪ) and ζ potential (Ɪ) indicate the standard deviation obtained from three independent measurements (mean ± SD, n = 3). Capital letters, blue lowercase letters, and black lowercase letters indicate significant differences in D_H_, ζ potential and PDI of the samples, respectively (p < 0.05). (SSF: simulated salivary fluid, SGF: simulated gastric fluid, SIF: simulated intestinal fluid). (For interpretation of the references to colour in this figure legend, the reader is referred to the web version of this article.)

**Fig. 6:**
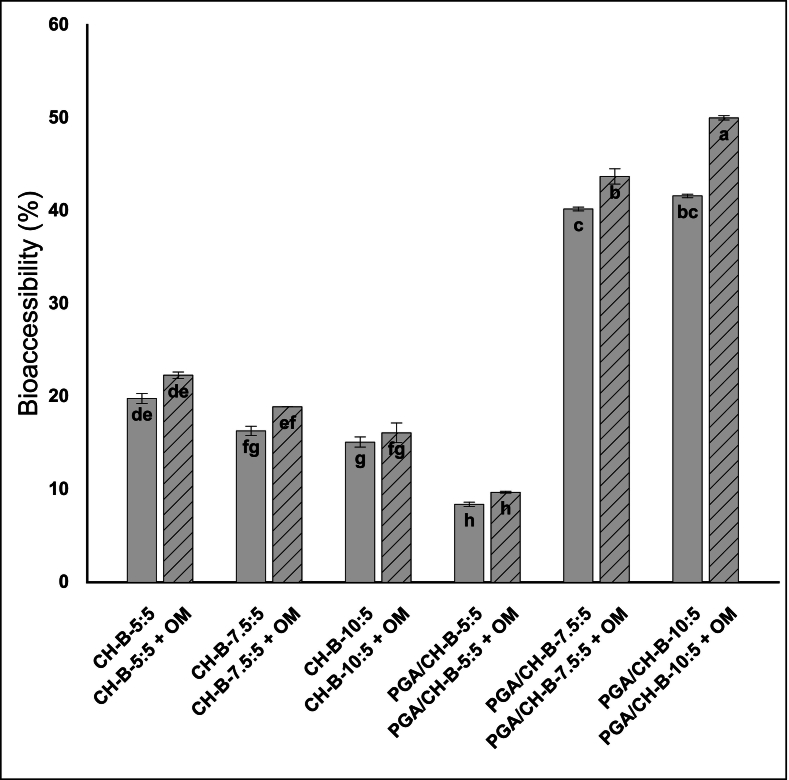
Concentration and bioaccessibility (%) of trans*-*resveratrol (*t*-res) in chitosan (CH)-coated and polygalacturonic acid (PGA)/CH-coated bilosomes digested without () and with oat milk (). Error bars for bioaccessibility (Ɪ) indicate the standard deviation obtained from three independent measurements (mean ± SD, n = 3). Letters indicate the significant differences in bioaccessibility of *t-*res from the samples (p < 0.05).

The initial properties (D_H_, PDI, ζ potential, and EE%) of *t*-res–loaded biopolymer-coated bilosomes are presented in Table 3, while *in vitro* digestion characteristics and bioaccessibility, after mixing with OM, are shown in Fig. 4, Fig. 5, Fig. 6.

**Table 3:** Properties of *t*-res loaded CH-coated and PGA/CH-coated bilosomes, without/with OM at pH 5.5.

	System	D_H_ (nm)	PDI	ζ potential (mV)	EE%
Without OM	CH-B-5:5	404.3 ± 5.0^de^	0.183 ± 0.025^ef^	17.4 ± 0.3^a^	98.0 ± 0.8^ab^
	CH-B-7.5:5	375.9 ± 4.4^de^	0.144 ± 0.01^f^	19.4 ± 0.9^a^	99.0 ± 0.4^a^
	CH-B-10:5	460.7 ± 21.0^d^	0.195 ± 0.021^ef^	21.5 ± 2.3^a^	98.4 ± 1.3^ab^
	PGA/CH-B-5:5	349.2 ± 7.4^e^	0.325 ± 0.011^bc^	−16.6 ± 0.9^b^	94.9 ± 0.2^ab^
	PGA/CH-B-7.5:5	451.4 ± 9.2^d^	0.467 ± 0.03^a^	−19.5 ± 2.4^b^	93.7 ± 1.5^b^
	PGA/CH-B-10:5	668.7 ± 22.0^b^	0.400 ± 0.019^ab^	−21.0 ± 1.9^b^	96.7 ± 0.4^ab^
With OM	CH-B-5:5	570.2 ± 10.3^c^	0.392 ± 0.035^ab^	−28.5 ± 0.5^d^	
	CH-B-7.5:5	603.3 ± 16.4^b^	0.405 ± 0.02^ab^	−27.1 ± 0.7^d^	
	CH-B-10:5	887.0 ± 73.4^a^	0.336 ± 0.029^bc^	−28.7 ± 0.6^d^	
	PGA/CH-B-5:5	389.5 ± 5.0^de^	0.255 ± 0.012^cd^	−25.6 ± 0.1^d^	
	PGA/CH-B-7.5:5	397.7 ± 3.1^de^	0.248 ± 0.024^de^	−25.0 ± 0.9^c^	
	PGA/CH-B-10:5	387.5 ± 3.1^de^	0.238 ± 0.026^e^	−25.9 ± 0.3^d^	

OM: Oat milk; D_H_: Hydrodynamic diameter; PDI: Polydispersity index; ζ: Zeta; EE%: Encapsulation efficiency. Data are presented as the mean ± standard deviation of three independent measurements (n = 3). Different superscript letters indicate statistically significant differences between samples within the same column (p < 0.05).

***CH-coated and PGA/CH-coated bilosomes:*** Bilosomes were first coated with CH *via* electrostatic interaction (Balanč et al., 2015), which effectively reversed the ζ potential from around −45 mV to +20 mV (*p* < 0.05), confirming successful surface modification (Table 3). The CH-coated bilosomes had D_H_ values of 375.9 ± 4.4 nm to 460.7 ± 21.0 nm, maintaining a low polydispersity (PDI ∼0.170), indicating uniform and stable dispersions. Except for the CH-B-10:5 formulation, the coating did not significantly affect D_H._ In CH-B-10:5, an increased CH may increase osmotic pressure, thereby promoting structural rearrangements within the bilosome system (Amenitsch et al., 2004). This effect is expected to be more pronounced in bilosomes with higher NaC concentrations, which exhibit enhanced membrane flexibility. In addition, higher CH may also facilitate the formation of thicker chitosan layers surrounding the vesicles. CH chain looping or potential flocculation phenomena at increased polymer concentrations may further contribute to an apparent increase in D_H_, as determined by DLS (Li et al., 2015).

EE% was high (>98%) across all formulations after chitosan coating and may result from the trapping of some free or loosely associated *t*-res within the coating layer, as well as from the protective effect of the biopolymer, which reduced degradation and leakage of encapsulated *t*-res during processing (Liu et al., 2015; Ramezanzade et al., 2017). Additionally, the biopolymer coating can promote the formation of multilayered vesicles, increasing the total volume available for drug encapsulation and thereby enhancing EE% (Amenitsch et al., 2004). Similarly, EE% of curcumin-loaded CH-coated liposomes reported as 84.0–99.8% (Chen et al., 2022). A subsequent coating with negatively charged PGA caused a second charge reversal, from around +20 mV back to around −20 mV, reflecting the anionic nature of the PGA coating (*p* < 0.05). The second coating yielded particles with a wider size range (D_H_: 349.2 ± 7.4 nm to 668.7 ± 22.0 nm) and higher PDI (∼0.400) (p < 0.05), reflecting broader size distributions and greater heterogeneity introduced by the final coating. Like CH-coated bilosomes, double-coated bilosomes exhibited high EE% values (93.7–96.7%) (*p* > 0.05).

Changes in *t*-res-loaded biopolymer-coated bilosomes **(**Fig. 4, Fig. 5**)**, after simulated digestion, were evaluated by D_H_, PDI, and ζ potential. In the oral phase, exposure to SSF caused aggregation of CH-coated bilosomes due to CH deprotonation at neutral pH (pKa ∼6.5), which lowers its positive charge. The reduced ζ potential weakens electrostatic repulsion, promoting flocculation (Sebaaly et al., 2021). In contrast, double-coated bilosomes showed minimal changes in D_H_, PDI, and ζ potential after SSF exposure, as PGA remains largely deprotonated at this pH, maintaining stability. In SGF (pH 3), CH-coated bilosomes displayed reduced D_H_, an increase in the magnitude of ζ potential, and improved homogeneity (*p* < 0.05), remaining stable throughout the gastric phase. This size reduction is likely due to protonation of amine groups of CH, under acidic conditions, which enhances electrostatic repulsion between particles, promoting redispersion of gastric-induced aggregates (Liu et al., 2016).

Similarly, when the oral bolus of double-coated bilosomes was exposed to SGF, both D_H_ and the magnitude of ζ potential decreased within the 5 min (*p* < 0.05), as PGA carboxyl groups became mostly protonated, yielding a near-neutral charge and a compact matrix *via* hydrogen bonding (Liu et al., 2016). Upon exposure to SIF, both CH-coated and double-coated bilosomes exhibited an increase in D_H_ accompanied by a rise in the magnitude of ζ potential (p < 0.05). For CH-coated bilosomes, the rapid size increase shortly after SIF exposure was likely due to CH deprotonation and loss of positive charge, forming a larger, less soluble biopolymer network. In double-coated bilosomes, the continued increase in ζ potential throughout the intestinal phase was attributed to PGA deprotonation and free fatty acid release, which increased negatively charged carboxylates, enhanced electrostatic repulsion, and expanded the biopolymer shell (Liu et al., 2016). This behaviour aligns with Liu et al., who reported a similar ζ potential shift in alginate/CH-coated liposomes, from −7.5 mV post-gastric to −20 mV early in the intestinal phase and reaching −55 mV after 120 min (Liu et al., 2013).

CH-coated bilosomes displayed pH- and ionic strength-responsive swelling/deswelling during digestion yet retained ∼85% of *t*-res (Fig. 6). Bioaccessibility of *t*-res was much lower (from 15.1 ± 0.6% to 19.8 ± 0.5%) compared to liposomes and bilosomes (*p* < 0.05), likely due to the TPP cross-linked CH shell, which protects against enzymes, bile salts, and pH shifts, slowing release of *t*-res (Hua et al., 2021). Higher CH concentrations further reduced *t*-res bioaccessibility (p < 0.05) by forming a thicker barrier that hindered release. Such sustained release is advantageous for colon-targeted delivery, where delay is needed until colonic fermentation. Comparable findings were reported by Andishmand et al., with only 33% of resveratrol released during oral and gastric phases, and Barea et al. reported ∼90% retention of 5-aminosalicylic acid in CH/Eudragit S100-coated liposomes *versus* 60% in non-cross-linked CH coatings (Andishmand et al., 2016; Barea et al., 2012). The PGA/CH-B-5:5 had even lower bioaccessibility (8.4 ± 0.2%, p < 0.05), indicating enhanced protection from the PGA layer. However, increasing the polymer concentration (PGA/CH-B-7.5:5 and PGA/CH-B-10:5) led to a sharp increase in bioaccessibility (∼40%), possibly due to osmotic pressure from excess polymer destabilizing the bilayer and promoting the leak of encapsulated compounds (Tan et al., 2021).

***CH-coated and PGA/CH-coated bilosomes co-digested with OM:*** When CH-coated bilosomes were mixed with OM, the D_H_ and PDI increased significantly **(**Table 3**)**, and ζ potential shifted from positive to around −28 mV (p < 0.05). The shift to a negative ζ potential indicates adsorption or association of negatively charged OM components, likely phospholipid/protein-coated oil droplets, onto the CH-coated bilosome surface (Zhao et al., 2021). This interaction led to increased D_H_ and PDI, reflecting aggregation. When PGA/CH-coated bilosomes were mixed with OM, D_H_ was largely unchanged except for PGA/CH-B-10:5, while PDI decreased to ∼0.250 and the magnitude of the ζ potential increased to around −25 mV (p < 0.05), suggesting enhanced electrostatic repulsion and improved colloidal stability. Changes in *t*-res-loaded biopolymer-coated bilosomes co-digested with OM (Fig. 4, Fig. 5) were assessed by D_H_, PDI, and ζ potential measurements. In the oral phase (SSF), D_H_ remained stable for both biopolymer-coated bilosomes-OM mix, but the magnitude of ζ potential decreased for CH-coated samples (p < 0.05). After mixing OM with CH-coated samples (pH ∼7.5), OM proteins carried negative charges while CH was weakly positive. In SIF, a slight pH drop made OM less negative and CH more positive, resulting in a reduced ζ potential magnitude (Sebaaly et al., 2021). In SGF, CH-coated bilosomes–OM mix showed no early changes, but D_H_ increased by the end of the gastric phase (p < 0.05) while ζ potential remained stable, likely from oil droplet coalescence and protein aggregation after pepsin hydrolysis as mentioned before (Mackie & Macierzanka, 2010; Zheng et al., 2021). PGA/CH-coated bilosomes displayed a marked increase in both D_H_ and the magnitude of ζ potential (p < 0.05), likely attributed to PGA protonation at low pH. This promotes aggregation or gel formation with hydrogen bonding with positively charged OM proteins (Liu et al., 2016). In addition, an excess concentration of PGA may lead to depletion flocculation (McClements, 2010). In SIF, both biopolymer-coated bilosomes-OM mixtures showed reduced D_H_ (∼500 nm) and PDI (∼0.300) along with a decreased magnitude of ζ potential, remaining stable for the rest of the phase. Although PGA had a more negative charge at pH 7 and the system was more homogeneous than the gastric phase, the magnitude of ζ potential decreased, possibly because of the buffering capacity of OM and the ionic screening effect by salts in SIF (Singh & Sarkar, 2011).

For CH-coated and PGA/CH-B-5:5 bilosomes, adding OM did not significantly affect *t*-res bioaccessibility **(**Fig. 6**)**, which remained below 20% without OM, indicating strong resistance to pH shifts, digestive enzymes, and bile salts. In contrast, higher PGA concentrations (PGA/CH-B-7.5:5 and PGA/CH-B-10:5) showed significant increases in bioaccessibility upon co-digestion with OM, by ∼3% and ∼ 8%, respectively (p < 0.05), likely due to leakage of the encapsulated compound.

### 3.5 Study limitations and future perspectives

Despite promising results, several limitations warrant consideration. The high cost of synthetic lipids such as POPC and DOPG suggests that natural alternatives may be preferable for large-scale applications. Further investigation into digestible biopolymer coatings is needed to clarify their impact on lipid bilayer integrity and targeted compound absorption. While the static *in vitro* digestion model provided insights into sample behaviour, OM effects, and *t*-res bioaccessibility, it cannot fully mimic *in vivo* dynamics, and the absence of mucin, salivary α-amylase, and gastric lipase may have influenced bilosome behaviour. *Ex vivo* absorption studies were limited by tissue availability, though using the same animal minimised variation. Addressing these limitations will enhance understanding of bilosome-based delivery systems for functional foods.

## 4 Conclusion

This study systematically examined the *in vitro* digestion behaviour, bioaccessibility, and intestinal absorption of *t*-res encapsulated in liposomes, bilosomes, and biopolymer-coated bilosomes, both in the presence and absence of OM as a food matrix. Incorporation of NaC was associated with changes in vesicle structure and improved encapsulation performance, stability, and bioaccessibility, with bilosomes generally outperforming free *t*-res and conventional liposomes. Bilosomes also showed higher intestinal absorption and tissue accumulation, whereas OM reduced tissue uptake, highlighting the influence of food matrix interactions. Although biopolymer coatings reduced immediate bioaccessibility, they improved vesicle integrity during upper gastrointestinal digestion, suggesting a potential for delayed release. Overall, these findings support the applicability of bilosomes as promising oral delivery systems for functional food formulations, while highlighting that their performance is formulation-dependent and influenced by the surrounding food matrix.

## CRediT authorship contribution statement

**Aygul Can:** Writing – original draft, Methodology, Formal analysis, Data curation, Conceptualization. **Taskeen Niaz:** Writing – review & editing, Methodology, Formal analysis, Data curation. **Arwen I.I. Tyler:** Writing – review & editing, Validation, Supervision, Project administration, Conceptualization. **Alan R. Mackie:** Writing – review & editing, Validation, Supervision, Project administration, Conceptualization.

## Ethics statement

Ethical approval was not applicable for this study.

## Declaration of competing interest

The authors declare the following financial interests/personal relationships which may be considered as potential competing interests: Given his role as Guest Editor, Alan Robert Mackie had no involvement in the peer-review of this article and has no access to information regarding its peer-review.

## Data Availability

Data will be made available on request.

## Appendix A Supplementary data

Supplementary information. Image 10
