# Supplementary material for: Impact of bile salts, biopolymer coatings, and food matrix on bilosome-mediated delivery of trans-resveratrol
Source: Food Chem X. 2026 Jan 16;34:103547. doi: 10.1016/j.fochx.2026.103547 (PMC12857395; doi:10.1016/j.fochx.2026.103547)
Supplement: Supplementary file 1 — Supplementary information. [file mmc1.docx]

**SUPPLEMENTARY INFORMATION**

**The enhanced bioavailability of trans-resveratrol using bilosome-based oral delivery system for food application**

**Table S1** Nutritional composition of the oat milk (Alpro) as given on the package labelling.

| **Ingredients** | Oat base (Water, Oat (8.7%)), Sunflower oil, Soluble corn fibre, Sugar, Pea protein, Calcium (Calcium carbonate), Acidity regulator (Potassium phosphates), Flavourings, Sea salt, Stabiliser (Gellan gum), Potassium Iodide, Vitamin D2. |
| --- | --- |
| **Nutritional information (per 100ml)** | Energy: 260 kJ / 62 kcal, Fat: 3.5 g (Saturated: 0.4 g, mono-unsaturated: 1 g and polyunsaturated: 2.1 g), Carbohydrate: 6.4 g (Sugars: 1.2 g), Fibre: 1 g, Protein: 0.7 g, Salt:0.12 g, Vitamin D: 0.75 µg, Calcium: 120 mg |

**Table S2** Composition of *t*-res loaded liposomes, bilosomes, and CH- and PGA/CH-coated bilosomes.

| **System** | **POPC (mM)** | **DOPG**  **(mM)** | **NaC (mM)** | **t-res (mM)** | **NaC/CH (w/w)** | **CH/PGA (w/w)** |
| --- | --- | --- | --- | --- | --- | --- |
| **L-5** | 10 | 3.3 | - | 5 | - | - |
| **B-5:5** | 10 | 3.3 | 5 | 5 | - | - |
| **B-7.5:5** | 10 | 3.3 | 7.5 | 5 | - | - |
| **B-10:5** | 10 | 3.3 | 10 | 5 | - | - |
| **CH-B-5:5** | 10 | 3.3 | 5 | 5 | 0.5 | - |
| **CH-B-7.5:5** | 10 | 3.3 | 7.5 | 5 | 0.5 | - |
| **CH-B-10:5** | 10 | 3.3 | 10 | 5 | 0.5 | - |
| **PGA/CH-B-5:5** | 10 | 3.3 | 5 | 5 | 0.5 | 0.4 |
| **PGA/CH-B-7.5:5** | 10 | 3.3 | 7.5 | 5 | 0.5 | 0.4 |
| **PGA/CH-B-10:5** | 10 | 3.3 | 10 | 5 | 0.5 | 0.4 |
| POPC: 2-oleoyl-1-palmitoyl-sn-glycero-3-phosphocholine; DOPG: 1,2-dioleoyl-sn-glycero-3-phospho-(1'-rac-glycerol) (sodium salt); NaC: Sodium cholate; t-res: trans-resveratrol; CH: Chitosan; PGA: Polygalacturonic acid. | | | | | | |

**Table S3** Volumes of electrolyte stock solutions of digestion fluids for a volume of 400 mL diluted with water (1.25× concentrations) (Brodkorb et al., 2019; Minekus et al., 2014).

| Salt solution added | Stock  concentrations | | SSF (pH 7) | | SGF (pH 3) | | SIF (pH 7) | |
| --- | --- | --- | --- | --- | --- | --- | --- | --- |
|  |  |  | Stock added to prepare 0.4 L (1.25×) | Final salt concentration  in SSF | Stock added to prepare 0.4 L (1.25×) | Final salt concentration  in SGF | Stock added to prepare 0.4 L (1.25×) | Final salt concentration  in SIF |
|  | (g/L) | (M) | (mL) | (mM) | (mL) | (mM) | (mL) | (mM) |
| KCl | 37.3 | 0.5 | 15.1 | 15.1 | 6.9 | 6.9 | 6.8 | 6.8 |
| KH_2_PO_4_ | 68 | 0.5 | 3.7 | 3.7 | 0.9 | 0.9 | 0.8 | 0.8 |
| NaHCO3^a^ | 84 | 1 | 6.8 | 13.6 | 12.5 | 25 | 42.5 | 85 |
| NaCl | 117 | 2 | - | - | 11.8 | 47.2 | 9.6 | 38.4 |
| MgCl_2_(H_2_O)_6_ | 30.5 | 0.15 | 0.5 | 0.15 | 0.4 | 0.12 | 1.1 | 0.33 |
| (NH_4_)_2_CO_3_^*^ | 48 | 0.5 | 0.06 | 0.06 | 0.5 | 0.5 | - | - |
| HCl |  | 6 | 0.09 | 1.1 | 1.3 | 15.6 | 0.7 | 8.4 |
| CaCl_2_(H_2_O)_2_^b^ | 44.1 | 0.3 | 0.025 | 1.5 | 0.005 | 0.15 | 0.04 | 0.6 |
| SSF: Simulated salivary fluid, SGF: Simulated gastric fluid, and SIF: Simulated intestinal fluid. ^a^ The use of carbonate salts in the electrolyte solutions requires the use of sealed containers with limited headspace, ^b^ CaCl_2_(H_2_O)_2_ should be added immediately before the digestion experiment to avoid precipitation during incubation. Volumes in **Table S3** are indicated for a typical experiment of 5 mL of SSF. The addition of enzymes, bile salts, Ca^2+^ solution etc. and water will result in the correct electrolyte concentration in the final digestion mixture. | | | | | | | | |

**Table S4** The effect of exposure to different phases of the static *in vitro* gastrointestinal digestion model on the PDI of t-res loaded samples digested without and with OM.

| **Sample** | | **initial** | **SSF** | **SGF** | | **SIF** | |
| --- | --- | --- | --- | --- | --- | --- | --- |
|  |  |  | **2 min** | **5 min** | **120 min** | **5 min** | **120 min** |
| ***t*-res** | **Without OM** | - | 0.794±0.007  a | 0.277±0.029  bcd | 0.207±0.019  d | 0.269±0.009  cd | 0.332±0.009  bc |
|  | **With OM** | 0.300±0.011  b | 0.093±0.053  e | 0.816±0.024  a | 0.916±0.041  a | 0.221±0.006  cd | 0.236±.004  cd |
| **L-5** | **Without OM** | 0.225±0.046  c | 0.202±.006  c | 0.242±.012  bc | 0.239±0.015  c | 0.251±0.022  bc | 0.233±0.004  c |
|  | **With OM** | 0.426±0.007  a | 0.133±0.069  c | 0.169±0.031  c | 0.154±0.079  c | 0.377±0.045  ab | 0.269±0.021  bc |
| **B-5:5** | **Without OM** | 0.259±0.048  bc | 0.291±0.003  bc | 0.348±0.032  ab | 0.354±0.040  ab | 0.255±0.004  bc | 0.226±0.013  cd |
|  | **With OM** | 0.429±0.018  a | 0.116±0.058  de | 0.051±0.020  e | 0.112±0.023  de | 0.305±0.024  bc | 0.216±0.041  cd |
| **B-7.5:5** | **Without OM** | 0.254±0.015  bcde | 0.291±0.002  abcd | 0.381±0.050  abc | 0.399±0.055  ab | 0.242±0.012  cde | 0.226±0.004  de |
|  | **With OM** | 0.419±0.010  a | 0.109±0.087  e | 0.234±0.025  cde | 0.111±0.078  e | 0.303±0.023  abcd | 0.250±0.008  bcde |
| **B-10:5** | **Without OM** | 0.264±0.023  b | 0.128±0.040  de | 0.229±0.013  bcd | 0.248±0.003  bc | 0.227±0.005  bcd | 0.265±0.016  b |
|  | **With OM** | 0.402±0.004  a | 0.157±0.031  cde | 0.280±0.065  b | 0.060±0.027  e | 0.318±0.029  ab | 0.230±0.018  bcd |
| *t*-res: trans-resveratrol, OM: oat milk, SSF: simulated salivary fluid, SGF: simulated gastric fluid, SIF: simulated intestinal fluid. Data are presented as the mean±standard deviation of three independent measurements (n=3). Lowercase letters indicate the significant differences between columns (in PDI of the samples during digestion) (p<0.05). | | | | | | | |


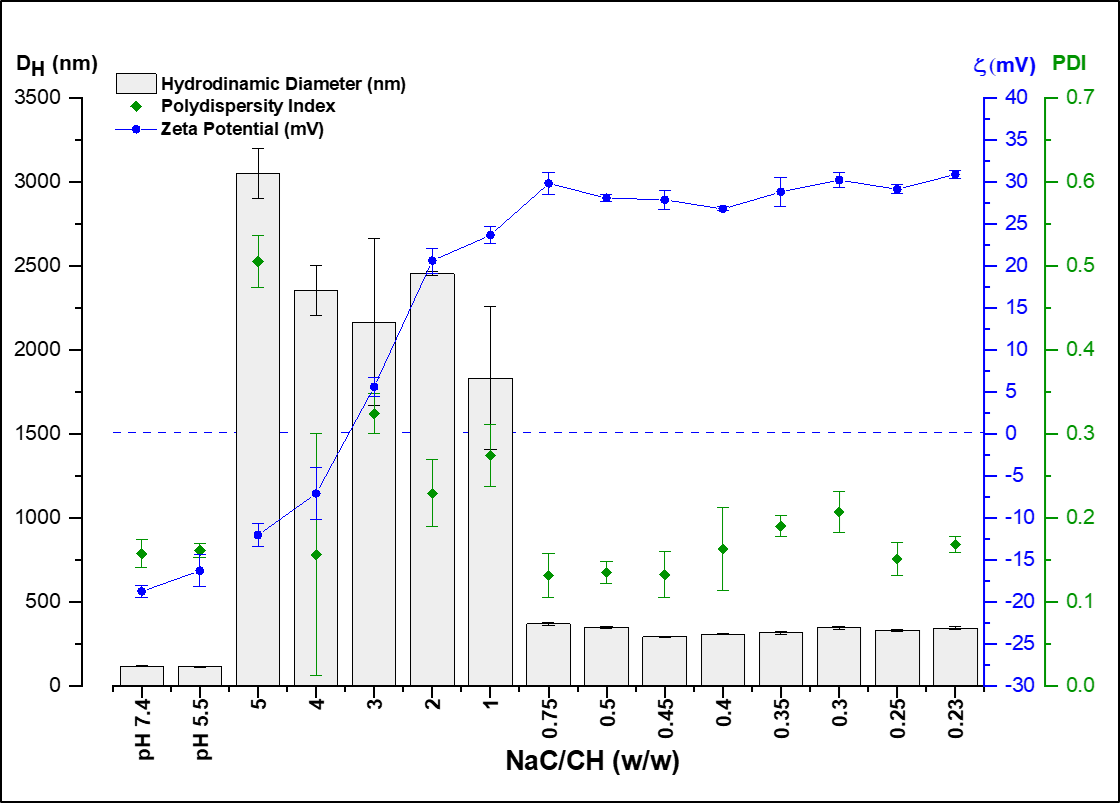


**Figure S1** Effect of NaC/CH (w/w) ratios on the hydrodynamic diameter (D_H_, nm,
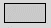
), polidisperdity index (PDI, ●) and zeta (ζ) potential (mV, ■) of B-5:0. Error bars for D_H_ (Ɪ), PDI (Ɪ), and ζ potential (Ɪ) indicate the standard deviation (SD) obtained from three independent measurements (mean±SD, n=3).

**
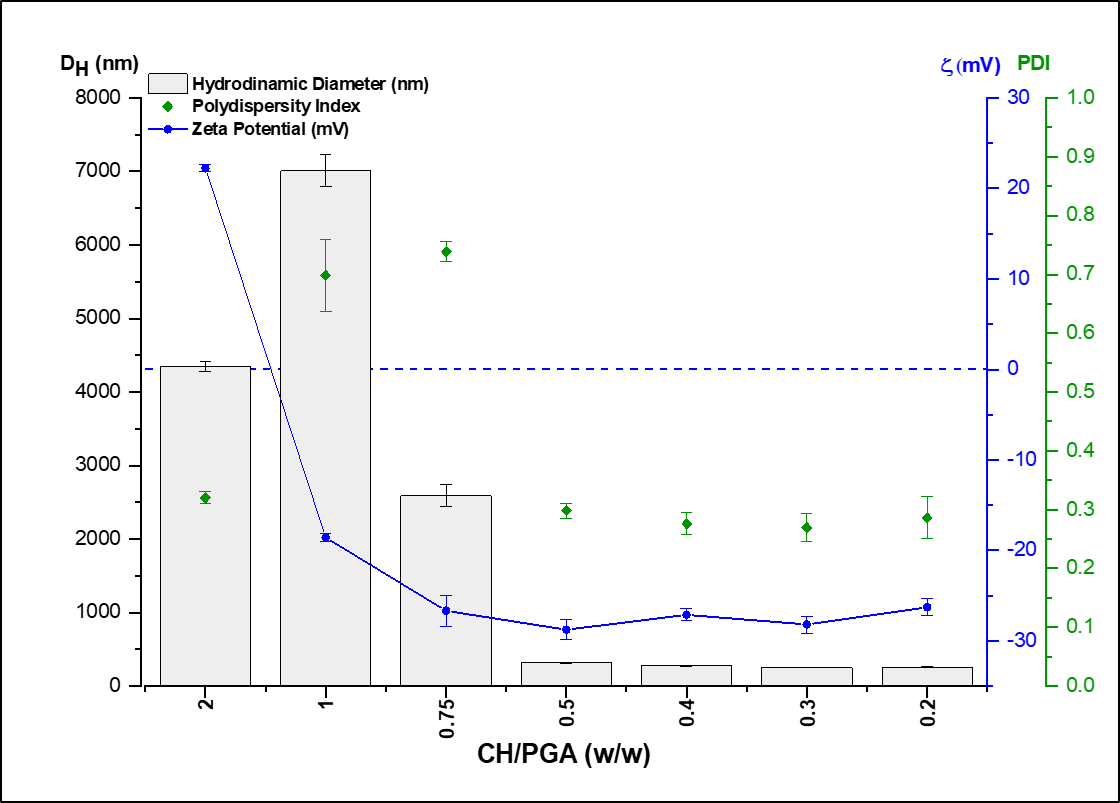
**

**Figure S2** Effect of CH/PGA (w/w) ratios on the hydrodynamic diameter (D_H_, nm,
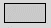
), polidisperdity index (PDI, ●) and zeta (ζ) potential (mV, ■) of B-5:0. Error bars for D_H_ (Ɪ), PDI (Ɪ), and ζ potential (Ɪ) indicate the standard deviation (SD) obtained from three independent measurements (mean±SD, n=3).


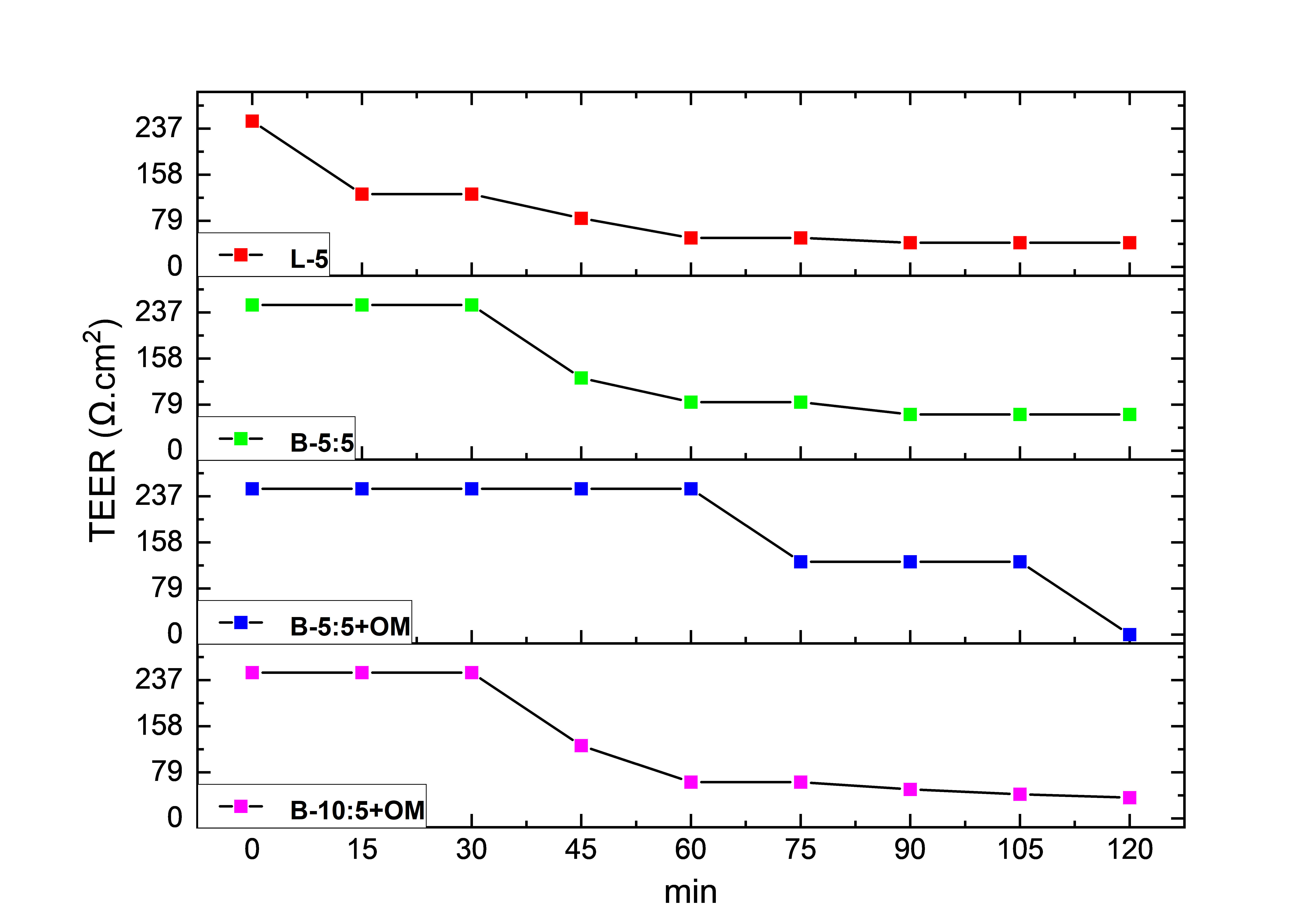


**Figure S3** Trans epithelial electrical resistance (TEER) value of murine intestinal tissue at 37°C during 120 min exposed to L-5 (◼), B-5:5 (◼), B-5:5+OM (◼), and B-10:5+OM (◼).


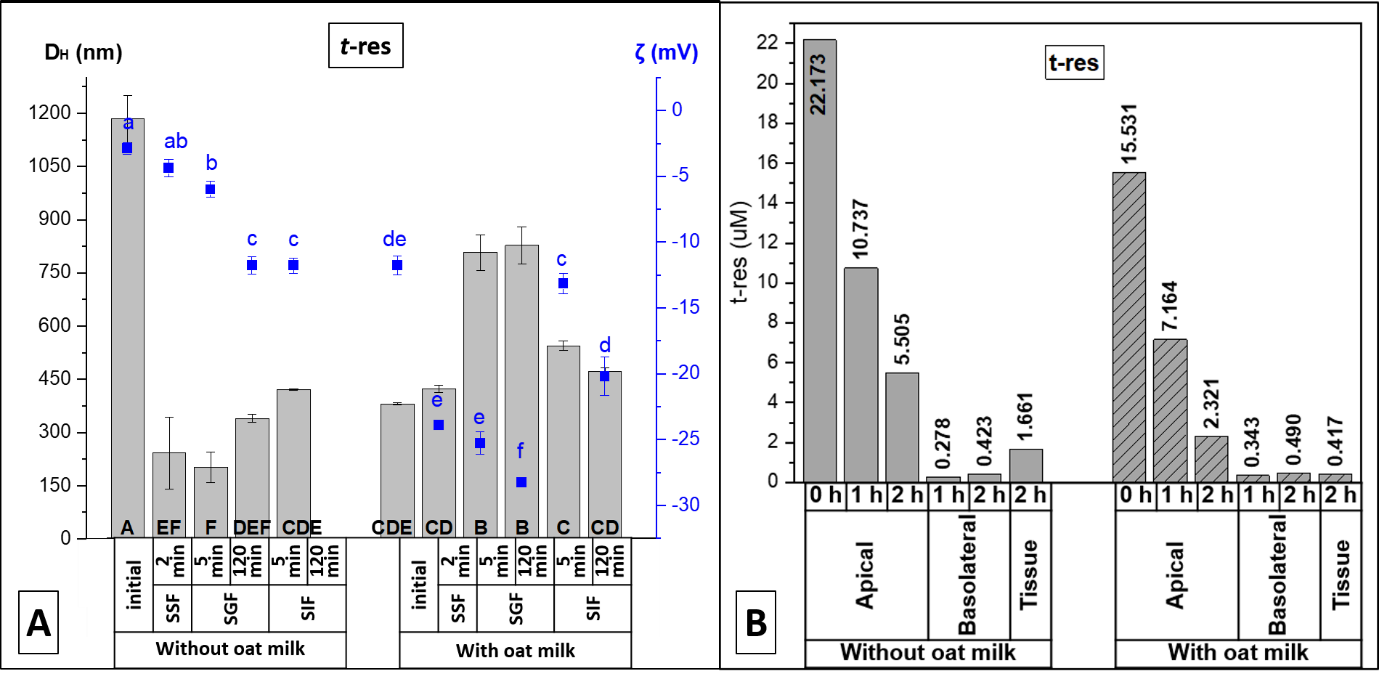


**Figure S4 (A)** Effect of exposure to different phases of the static *in vitro* gastrointestinal digestion model on the hydrodynamic diameter (D_H_, nm,
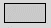
) and zeta (ζ) potential (mV, ■) of free *trans* resveratrol (*t*-res) without and with oat milk (OM). Error bars for D_H_ (Ɪ), and ζ potential (Ɪ) indicate the standard deviation (SD) obtained from three independent measurements (mean±SD, n=3). **(B)** Concentration changes of free *t-*res (
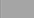
) and *t-*res digested with OM (
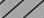
) in the apical and basolateral chambers after exposure to the murine intestinal mucosa, along with the concentration of accumulated *t-*res in the tissue. Capital letters and lowercase letters indicate the significant difference between in D_H_ and ζ potential of the samples, respectively (p<0.05). (SSF: simulated salivary fluid, SGF: simulated gastric fluid, SIF: simulated intestinal fluid).

**REFERENCES**

Brodkorb, A., Egger, L., Alminger, M., Alvito, P., Assunção, R., Ballance, S., Bohn, T., Bourlieu-Lacanal, C., Boutrou, R., & Carrière, F. (2019). INFOGEST static in vitro simulation of gastrointestinal food digestion. *Nature protocols*, *14*(4), 991-1014.

Minekus, M., Alminger, M., Alvito, P., Ballance, S., Bohn, T., Bourlieu, C., Carriere, F., Boutrou, R., Corredig, M., Dupont, D., Dufour, C., Egger, L., Golding, M., Karakaya, S., Kirkhus, B., Le Feunteun, S., Lesmes, U., Macierzanka, A., Mackie, A., . . . Brodkorb, A. (2014). A standardised static in vitro digestion method suitable for food - an international consensus. *Food Funct*, *5*(6), 1113-1124. <https://doi.org/10.1039/c3fo60702j>
